# Transcriptional Activation of p53 during Cold Induced Torpor in the 13-Lined Ground Squirrel *Ictidomys tridecemlineatus*

**DOI:** 10.1155/2015/731595

**Published:** 2015-12-30

**Authors:** Joshua Hefler, Cheng-Wei Wu, Kenneth B. Storey

**Affiliations:** Institute of Biochemistry and Department of Biology, Carleton University, 1125 Colonel By Drive, Ottawa, ON, Canada K1S 5B6

## Abstract

The transcription factor p53 is located at the centre of multiple pathways relating the cellular response to stress. Commonly known as a tumor suppressor, it is responsible for initiating diverse actions to protect the integrity of the genome, ranging from cell cycle arrest to apoptosis. This study investigated the regulation of p53 protein in hibernating 13-lined ground squirrel* Ictidomys tridecemlineatus* during multiple stages of the torpor-arousal cycle. Transcript and protein levels of p53 were both elevated in the skeletal muscle during early and late torpor stages of the hibernation cycle. Nuclear localization of p53 was also increased during late torpor, and this is associated with an increase in its DNA binding activity and expression of p53 transcriptional targets* p21CIP*,* gadd45α*, and* 14-3-3σ*. The increase in p53 transcriptional activity appears to be independent of its phosphorylation at Ser-15, Ser-46, and Ser-392, consistent with an absence of checkpoint kinase activation during torpor. Sequence analysis revealed unique amino acid substitutions in the ground squirrel p53 protein, which may contribute to an increase in protein stability compared to nonhibernators. Overall, the study results provided evidences for a potential role of p53 in the protection of the skeletal muscle during torpor.

## 1. Introduction

Hibernation is an adaptive strategy characterized by dramatic changes in the physiology, behaviour, and biochemistry of the animal in response to increased environmental stress. This phenotypic plasticity is found among diverse mammalian groups, even stretching as far back in the lineage as monotremes and marsupials [1]. Rodentia contains some of the most well studied hibernators, including the subject of this study, the 13-lined ground squirrel (*Ictidomys tridecemlineatus*).* I. tridecemlineatus*, an inhabitant of the central North American grasslands, typically hibernates from October to April, when seasonal changes that increase metabolic demand are met with a restriction on food supply. Hibernation in* I. tridecemlineatus* is characterized by cyclic arousal and reentry into a low metabolic state [1]. Physiologically, the animal experiences a decrease in body temperature (from 37°C to as low as 1-2°C), heart rate (from 200–300 to 3–5 beats/min), perfusion rate (<10% of normal), and respiration rate (from 100–200 to 4–6 breaths/min) upon entry into torpor [1–3]. Overall, metabolism can be decreased to 2–4% of its euthermic rate [4]. Despite these drastic changes,* I. tridecemlineatus* emerges from torpor experiencing little to no ill effects, including limited signs of muscle atrophy despite months of inactivity [5].

While the physiological and behavioural changes of hibernation have been studied for some time, little is known about the underlying molecular mechanisms. With a lowered metabolic rate comes a global decrease in transcription and translation [2, 3]. Genes that continue to be transcribed and translated will likely hold key to understanding hibernation from a cellular perspective. One gene, whose regulation during hibernation may prove to be essential, is p53. The p53 protein is a well-known transcription factor due to the prevalence of its mutant in cancers (>50%), but the source of its antitumorigenic properties lies within its integration into multiple stress-responsive pathways [6]. Stressors such as hypoxia, heat or cold shock, DNA damage, or nutrient deprivation can activate p53 by posttranslation modification (i.e., phosphorylation, methylation, and acetylation), resulting in its transcriptional activation or repression of numerous downstream target genes [7]. These transcriptional programs can lead to diverse cellular responses, most notably cell cycle arrest, senescence, and apoptosis [6, 7]. Cell cycle arrest in particular largely involves p53 downstream targets that cause either the dissociation or nuclear exclusion of cyclin/Cdk complexes, which promote events necessary for cell division (e.g., DNA replication, centrosome duplication, and mitotic spindle formation) [8, 9]. An important regulator of p53 is MDM2, which in addition to being a transcriptional target of p53 promotes its degradation through the ubiquitin-proteasome pathway [10].

Given that hibernating ground squirrels are able to undergo dramatic physiological changes that would prove highly stressful and burdensome to nonhibernating animals, while being able to maintain the integrity and functionality of their tissues and organs, it is reasonable to suspect the involvement of p53 given its role in preservation of cells and conservation of energy through cell cycle arrest. This study examined the changes in transcript and protein levels of p53 and several of its downstream targets in the skeletal muscle of* I. tridecemlineatus* throughout hibernation. We show that p53 expression levels and transcriptional activities are elevated during late torpor, and this is regulated at the translational level and independent of posttranslational modifications. We also identify several ground squirrel specific amino acid substitutions that may contribute to an overall increase in p53 stability that could assist in transcriptional activation during torpor.

## 2. Materials and Methods

### 2.1. Animals

Thirteen-lined ground squirrels (*Ictidomys tridecemlineatus*) were captured by a licensed trapper (TLS Research, Michigan) and delivered to the laboratory of Dr. J. M. Hallenbeck (National Institute of Neurological Disorders and Stroke, NIH, Bethesda, MD). The animal treatment protocols were the same as previously described in detail [11]. The squirrels were sacrificed by decapitation at one of six different time points. For the euthermic control condition (EC), squirrels were sacrificed after being active at 4°C for 3 days without having entered a new torpor bout. In other conditions, squirrels were sacrificed during entry into torpor (EN), with body temperature (*T*_*b*_) fallen to 18–31°C, after 1-2 days in torpor (ET) with *T*_*b*_ stable at 5–8°C, after 3–5 days in torpor (LT) with *T*_*b*_ stable at 5–8°C, during early arousal (EA) with *T*_*b*_ rising to 9–12°C, and during interbout arousal (IA) when *T*_*b*_ was restored to ~37°C for at least 18 h. Tissues were excised, stored in liquid nitrogen, and transported to Carleton University on dry ice, where they were stored at −80°C until use. All squirrels were cared for in accordance with the Animal Welfare Act and all animal experiments received prior approval from the NIH.

### 2.2. Total RNA Extraction, cDNA Synthesis, and RT-PCR

Prior to use, all materials were treated with 0.1% (v/v) diethylpyrocarbonate (DEPC) and autoclaved. Total RNA was isolated using standard Trizol techniques from ground squirrel muscle tissue for each of the six time points, with four independent samples prepared for each time point. RNA quality was assessed using the ratio of absorbance at 260 and 280 nm, in addition to electrophoresis on a 1.5% agarose gel to determine the integrity of the 18S and 28S ribosomal bands.

Aliquots of 3 *μ*g of RNA from each sample were diluted with 7 *μ*L of DEPC water. One *μ*L of 200 ng/*μ*L oligo-dT primer was added to each sample, followed by incubation at 65°C for 5 min. Four *μ*L of 5x First strand buffer, 2 *μ*L of 0.1 M DTT, 1 *μ*L of 10 mM dNTP (BioShop), and 1 *μ*L of M-MVL reverse transcriptase (Invitrogen) were added to each tube. The samples were incubated at 42°C for 45 min for cDNA synthesis. A 10^−1^ and 10^−2^ dilution of the resulting cDNA were created and stored at −20°C until use. Polymerase chain reaction (PCR) was carried out as previously described [12]. Primers used for this study were as follows: 5′-CCTGCTGATGGAACGTCTCT-3′ and 5′-GTAAGCTGTTCATGGTAGGC-3′ (*α-tubulin*), 5′-GAGGTCGGCTCTGACTATACC-3′ and 5′-ATTCAGCTCTCGGAACATCTC-3′ (*p53*), 5′-AACCTGCTCTCCGTGGCCTAC-3′ and 5′-CTCGTCGAAGGTGGTCTTGG-3′ (*14-3-3σ*), 5′-GCCAAGCTGCTCAACGTAGA-3′ and 5′-GATGTTGATGTCGTTCTCGC-3′ (*gadd45α*), and 5′-CAATCAGCAGGAACCGTCAG-3′ and 5′-GAGTCCTGATCCAACCAATC-3′ (*mdm2*).

### 2.3. Tissue Preparation and Western Blotting

Frozen tissue samples were homogenized in 1 : 5 (w/v) homogenization buffer (20 mM HEPES, pH 7.5, 200 mM NaCl, 0.1 mM EDTA, 10 mM NaF, 1 mM Na_3_VO_4_, and 10 mM *β*-glycerophosphate) supplemented with several crystals of PMSF and 1 *μ*L of protease inhibitor cocktail (Sigma-Aldrich). The supernatant was collected after samples were centrifuged at 4°C for 15 min at 10,000 ×g. Protein concentrations of the samples were determined via the Coomassie Blue dye-binding method using the BioRad prepared reagent, with bovine serum albumin used as a standard. Protein concentrations of samples were then adjusted to 10 *μ*g/*μ*L with homogenization buffer and then diluted to 5 *μ*g/*μ*L by adding an equal amount of 2X SDS buffer (100 mM Tris-base, 4% w/v SDS, 20% v/v glycerol, 0.2% v/v bromophenol blue, and 10% v/v *β*-mercaptoethanol). The samples were heated for 5 min at 95°C and stored at −20°C until use.

Western blot procedures were carried out as previously described [13]. Antibodies used for this study were as follows: p53 (#2527, Cell Signaling), phospho-p53 (Ser15; #9284, Cell Signaling), acetyl-p53 (Lys382; #2525, Cell Signaling), phospho-p53 (Ser392; #9281, Cell Signaling), phospho-p53 (Ser46; #2521, Cell Signaling), phospho-Chk1 (Ser296, #A00727-40, Genscript), phospho-Chk2 (Thr68, GTX61178, Gentex), MDM2 (#SC-813, Santa Cruz Biotechnology), and GAPDH (#2118, Cell Signaling).

### 2.4. Nuclear Extracts

Nuclear extracts were prepared for the skeletal muscle from EC and LT time points using a modified version of the method described by Dignani et al. [14]. Approximately 0.5 g of tissue samples was homogenized in 1 mL of homogenization buffer (10 mM HEPES, 10 mM KCl, 10 mM EDTA, 1 mM DTT, pH 7.9) with the addition of 10 *μ*L of Protease Inhibitor Cocktail (Sigma-Aldrich). The samples were centrifuged at 10,000 ×g for 10 min at 4°C. The supernatant containing the cytoplasmic fraction was removed and stored for later use. Remaining tissue pellets were resuspended in 150 *μ*L of nuclear extraction buffer (20 mM HEPES, 400 mM NaCl, 1 mM EDTA, 10% v/v glycerol, and 1 mM DTT) with 1.5 *μ*L of Protease Inhibitor Cocktail (Sigma-Aldrich) and incubated for 1 h on ice while rocking. The resuspension was centrifuged at 10,000 ×g for 10 min at 4°C. The supernatant containing the nuclear fraction was then collected. A portion of nuclear fraction was stored at −20°C for use in the DNA binding enzyme-linked immunosorbent assay (ELISA), while the remaining nuclear fractions were treated as described above for use in Western blotting. Western blotting using the primary antibody for histone H3 (#9715, Cell Signaling) confirmed the purity of the nuclear extracts.

### 2.5. DNA Binding ELISA

The concentrations of the nuclear extracts prepared as described above were adjusted to 10 *μ*g/*μ*L with the nuclear extraction buffer. Wells of a 96-well microplate coated with streptavidin were probed for 1 h at RT with double stranded biotin-labelled DNA probe containing the binding sequence of p53: 5′-TACCCGGGCATGTGCTAAGCATGCTG-3′, 3′-CAGCATGCTTAGACATGCCCGGGTA-5′. After washing three times with PBST (PBS with 0.05% Tween-20), each well was incubated with 20 *μ*g of protein sample for 1 h at RT (~24°C) or at 5°C on ice. This was followed by washing and probing with p53 primary antibody (1 : 1000 in PBST) for 1 h at RT with agitation. After washing, the wells were probed with HRP-linked secondary antibody (1 : 4000 in PBST) for 1 h at RT with agitation. After four times washing with PBST, a solution containing 3,3′,5,5′-tetramethylbenzidine was added to each well for p53 binding detection. Reaction was stopped with the addition of 1 M HCl, and absorbance read at 450 nm (with reference at 655 nm) was recorded and used to calculate relative p53 DNA binding between EC and LT samples.

### 2.6. Statistics

Western blots were imaged and their relative intensities were quantified using the ChemiGenius Bio-Imaging System with the GeneTools program (SynGene). Relative protein levels were normalized to the housekeeping protein GAPDH while mRNA levels were normalized to *α-tubulin*. Data were plotted as means ± SEM and tested for significance with Student's *t*-test (two data points) or a one-way analysis of variance (three or more data points; Holm-Sidak test) to assess significant difference, with *p* < 0.05 accepted as statistically significant.

### 2.7. Sequence Analysis of p53 Protein

Protein sequences of p53 were retrieved for* Ictidomys tridecemlineatus* (Gene ID: 101957738),* Erinaceus europaeus* (Gene ID: 103113788),* Myotis brandtii* (Gene ID: 102240449),* Rattus norvegicus* (Gene ID: 24842),* Cricetulus griseus* (Gene ID: 100682525),* Homo sapiens* (Gene ID: 7157), and* Macaca fascicularis* (Gene ID: 102135998) from NCBI and aligned for sequence analysis using the Geneious software. Ground squirrel amino acids that had altered residues at positions that were conserved among the four other nonhibernating mammals were identified as unique p53 substitutions.

## 3. Results

### 3.1. Expression of p53 Is Elevated during Torpor, but Not Differentially Modified

The p53 protein has multiple sites for posttranslation modifications, including at least 18 sites for phosphorylation and at least 10 for acetylation [15]. It has been shown that posttranslational modifications are not necessary for overall p53 activation; however, phosphorylation and acetylation at different sites can stabilize p53 leading to subsequent transcriptional activation. The levels of p53 phosphorylated at serines 15, 46, and 392 and acetylated at lysine 382 were investigated in the skeletal muscle of* I. tridecemlineatus* during the six stages of torpor. In skeletal muscle, total p53 was increased by approximately twofold in both early (1.93 ± 0.02) and late (2.36 ± 0.07) torpor (*p* < 0.05). Interestingly, there were no significant changes in any of the posttranslationally modified p53 forms measured in this study. These results suggest that p53 may be activated during torpor through an upregulation in total p53 protein level.

### 3.2. Regulators of p53 Are Not Differentially Expressed during Torpor

**Figure 1:**
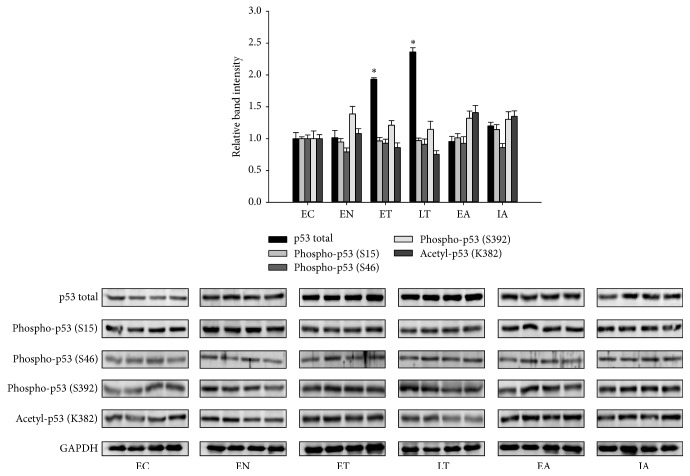
p53 protein expression and posttranslational modifications in the ground squirrel skeletal muscle over six stages of the torpor cycle. Stages of torpor are EC (euthermic in cold room), EN (entry into torpor), ET (early torpor), LT (late torpor), EA (early arousal), and IA (interbout arousal); see Section 2 for more details. Relative protein levels and Western blots are shown for total p53, p53 phosphorylated at serine 15, 46, and 392, p53 acetylated at lysine 382, and the housekeeping protein GAPDH. Data show means ± SEM, *N* = 4-5 independent trials on tissues from different animals. *∗* denotes significant statistical difference from EC values, *p* < 0.05.

**Figure 2:**
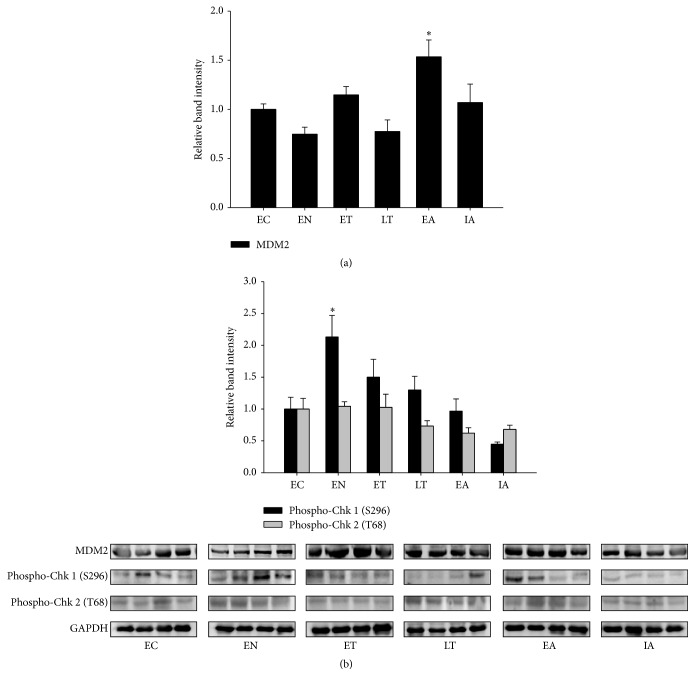
Expression of p53 upstream regulators in the ground squirrel skeletal muscle over six stages of the hibernation cycle. Relative protein expressions and Western blots are shown for (a) MDM2 protein, and (b) phosphorylated checkpoint kinase 1 (S296) and checkpoint kinase 2 (T68). Data show means ± SEM, *N* = 4-5 independent trials on tissues from different animals. *∗* denotes significant statistical difference from EC values, *p* < 0.05.

The MDM2 protein is E3 ubiquitin ligase that under normal and nonstressed conditions functions as a principal antagonist of p53 through continuous degradation by monoubiquitination [10]. The protein level of MDM2 was upregulated by 1.53 ± 0.17-fold during early arousal, while remaining unchanged at other torpor time points (Figure 2(a)). Under cellular stresses such as DNA damage and hypoxia, p53 can be posttranslationally modified to increase protein stabilization through disruption of the p53-MDM2 interaction. Checkpoint kinases (Chk) 1 and 2 are two serine/threonine protein kinases that are activated during DNA damage by phosphorylation and can in turn phosphorylate p53 at Ser-15 to alleviate its inhibition by MDM2 [16]. We measured the phosphorylation levels of Chk1 and Chk2 at its activating residues. Shown in Figure 2(b), phosphorylation of Chk1 (Ser-296) was increased by 2.13 ± 0.33-fold during entry into torpor, while remaining unchanged at other time points. Meanwhile, the phosphorylation levels of Chk-2 (Thr-68) remained unchanged throughout the torpor-arousal cycle. The lack of Chk1/2 kinase activation during the torpor stages is consistent with our observations of a stable p53 phosphorylation level throughout torpor (Figure 1). These results suggest that activation of p53 through the canonical DNA damage pathway likely does not take place during torpor in the skeletal muscle, and that the upregulation of MDM2 during early arousal may serve to renormalize the p53 protein levels as animals exit hibernation.

### 3.3. Nuclear Levels of p53 and DNA Binding Activity Are Increased during Torpor

**Figure 3:**
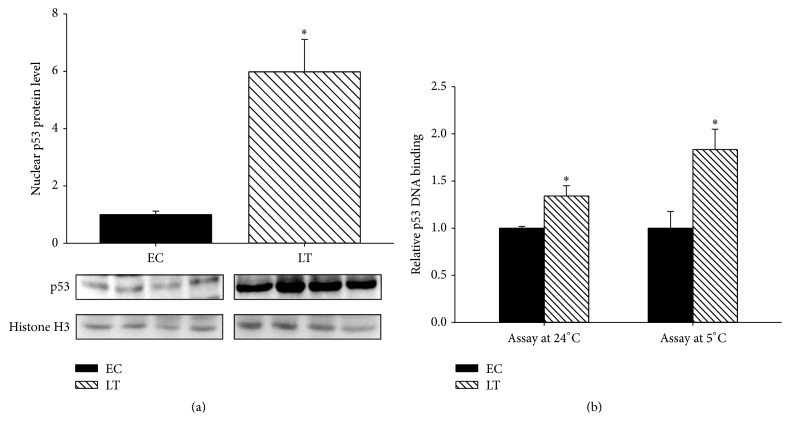
Nuclear regulation of p53 during late torpor. (a) Western blot is shown for nuclear levels of p53 and histone H3 during euthermic control and late torpor. (b) p53 DNA binding activity towards double stranded probe containing p53 consensus binding sequence (5′-GGACATGCCCGGGCATGTCC-3′) in control and late torpor nuclear lysates. Data show mean ± SEM, *N* = 4 independent trials on tissues from different animals. *∗* denotes significant statistical difference from EC values, *p* < 0.05.

For p53 to exert its function, it must be transported into the nucleus and bind to the promoters of target genes. The relative amount of p53 proteins in the nuclear lysates was compared under the control condition and late torpor in the skeletal muscle. Shown in Figure 3(a), nuclear levels of p53 were significantly elevated by nearly 6-fold (5.98 ± 1.12) during late torpor compared to euthermic control levels. This increase in nuclear p53 proteins during late torpor was also supported by an increase in p53 DNA binding activity during late torpor (Figure 3(b)). The p53 DNA binding activity during torpor was elevated by 1.3 ± 0.11-fold compared to the control; similar results were obtained when the binding assay was carried out 5°C, with late torpor lysates also showing a 1.8 ± 0.21-fold increase in p53 DNA binding activity.

### 3.4. Transcript Expressions of p53 and Its Targets Are Elevated during Torpor

To determine if the increase in p53 nuclear expression and DNA binding activity during late torpor was accompanied by an increase in transcription of p53 downstream genes, we measured the transcript levels of p53 downstream target genes* 14-3-3σ*,* gadd45α*, and* p21*, as well as the mRNA level of* p53*, and its principle inhibitor of* mdm2* in the skeletal muscle of* I. tridecemlineatus* (Figure 3). The mRNA transcript levels of* p53* were significantly elevated during early torpor (1.74 ± 0.29), late torpor (1.60 ± 0.23), and interbout arousal (2.05 ± 0.36), while* mdm2* transcripts were mostly stable throughout the torpor cycle. The transcript levels of p53 downstream gene* 14-3-3σ* were increased significantly in early and late torpor (2.16 ± 0.26 and 1.75 ± 0.15, resp.), while transcript levels of* gadd45α* were increased during late torpor (1.64 ± 0.16), and transcript levels of* p21* were increased during late torpor (1.74 ± 0.32) and early arousal (1.60 ± 0.13) compared to the control. These results suggest that the increase in total p53 protein levels during torpor was accompanied by an elevated* p53* mRNA level, and this in turn promotes the increase in nuclear p53 accumulation and subsequent transcription of its downstream genes.

### 3.5. Identification of Unique p53 Protein Sequence

The human p53 protein is the most common target for mutations in cancer, with more than 50% of all cancerous tumors exhibiting mutations at the p53 gene locus [17]. Although majority of the p53 mutations result in a nonfunctional protein, mutations that result in gain of functions have also been reported [18]. To determine if the ground squirrel p53 protein contains any unique amino acid substitutions that may contribute to its differential regulation, we aligned the ground squirrel p53 protein sequence to two hibernating and four nonhibernating mammals to identify potential unique amino acid substitutions. Shown in Figure 5 and Table 1, a total of 12 ground squirrel unique amino acid substitutions were identified at residues that are conserved among the four nonhibernating species. Of the 12 squirrel specific p53 amino acids, only 3 residues (Asn 24, Pro 299, and Pro 362) were conserved in at least one other hibernator (Brandt's bat,* Myotis brandtii,* or European hedgehog,* Erinaceus europaeus*). The substitution of Lys→Arg at position 290 (292 in human) and Ser→Pro at position 364 (366 in human) in ground squirrels is of particular interest, as previous studies have associated these two amino acid residues to be posttranslationally modified and involved in regulating p53 stability. The Lys-292 residues in human are acetylated and targeted by MKRN1 (Makorin Ring Finger Protein) to promote p53 degradation, and a mutation of Lys-292 to Arg was shown to significantly increase p53 stability through resistance towards MKRN-1 mediated degradation [19]. Meanwhile, phosphorylation of the Ser-366 residue in human by IkappaB kinase 2 (IKK2) leads to p53 ubiquitination and subsequent degradation, whereas amino acid substitution at Ser-366 to Ala results in an increase in p53 stability and its transcriptional activity [20]. These results suggest that unique changes in the ground squirrel p53 sequence could contribute to a potential increase in protein stability, which may assist in its elevated protein expression and transcriptional activity in the skeletal muscle during torpor.

## 4. Discussion

Hibernation is a hypometabolic state that is characterized by a global suppression of energy consumption in order to decrease metabolic demands to prolong survival. Although transcription is largely suppressed, multiple studies have shown that expressions of select genes are elevated during torpor to promote adaptations towards a hypometabolic state and are facilitated by many stress inducible transcription factors that include FOXO, Nrf2, and Nfk-*β* [21–23]. In this study we characterized the regulation of p53 in the skeletal muscle of winter ground squirrels during the torpor-arousal cycle, to determine if p53 is activated between the intermediate torpor stages of hibernation. A recent study by Pan et al. showed that p53 is activated in the liver tissues of ground squirrels during torpor compared to summer active squirrels, while p53 showed an increase in DNA binding activity in the liver; it did not lead to increase in transcription of p53 target genes [24]. Combined with our current results, this suggests that p53 is regulated in a tissue specific manner during torpor. In addition, the differences in p53 transcriptional activity observed by Pan et al. could also be partially attributed by the use of summer active squirrels as the control group, which exhibits seasonal differences in basal gene and protein expressions compared to hibernation ready winter squirrels used in the present study [25].

**Figure 4:**
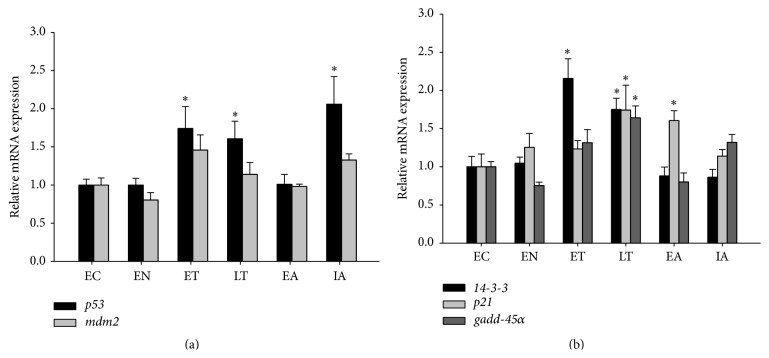
Transcriptional regulation of p53 in the hibernation cycle. Transcript levels of (a)* p53*,* mdm2,* and (b) p53 downstream targets* 14-3-3σ*,* p21*, and* gadd-45α* are shown in the ground squirrel skeletal muscle over six stages of the hibernation cycle. Transcript levels were normalized to the housekeeping gene *α-tubulin*. Data show means ± SEM, *N* = 4-5 independent trials on tissues from different animals. *∗* denotes significant statistical difference from EC values, *p* < 0.05.

The p53 protein is a transcription factor that is activated in response to multiple stressors that include hypoxia, heat shock, oxidative stress, and DNA damage [6]. The activation of p53 results in the transcription of genes that function to promote cell cycle arrest, apoptosis, and cellular senescence; however, recent studies have also suggested a role for p53 in regulation of cellular metabolism [26]. The p53 protein contains a large number of residues that are subject to posttranslational modifications that include phosphorylation and acetylation, with majority of these residues rapidly modified following cellular stress [15]. Interestingly, we observed no significant changes in the phosphorylation and acetylation state of p53 protein throughout the torpor-arousal cycle, and this was largely consistent with the absence of checkpoint kinase activation (Figure 2(b)). Checkpoint kinases 1 and 2 are activated by ataxia telangiectasia mutated (ATM) and ataxia telangiectasia and Rad3-related (ATR) protein by phosphorylation in response to DNA damage [27] and in turn phosphorylate p53 to initiate a transcriptional response; our data here suggest that p53 is not activated by DNA damage during torpor. Although canonical activation of p53 by phosphorylation was not observed, the total protein and mRNA expression of p53 was upregulated during both torpor stages (Figure 1) in absence of a similar increase in its principal repressor MDM2 (Figure 2(a)). This increase in total p53 protein content during the torpor was also associated with an increase in nuclear p53 protein content and DNA binding activity, along with an increase in transcription of p53 target genes (Figures 3 and 4). Although we observed a robust elevation in nuclear p53 accumulation during late torpor (~6-fold), this was accompanied by only a modest increase in p53 DNA binding activity and downstream gene expression (both by ~1.5- to 1.8-fold). Similar observations of insufficient recruitment of nuclear p53 to DNA were also reported by Pan et al., which showed that a 2-fold increase in nuclear p53 in ground squirrel livers during torpor only resulted in a 40% increase in p53 DNA binding [24]. These results would suggest that other cofactors and regulators are likely required for the binding and activation of nuclear p53 to target DNA, and the total levels of nuclear p53 levels may not be proportional to its transcriptional activity. Interestingly, although p53 protein was upregulated at both early and late torpor stages, the increase in target transcript levels was only largely observed during late torpor (Figure 4(b)). The transcript of* mdm2* itself is also a downstream target of p53, forming an autoregulatory feedback loop to control p53 activity [10]; however, the mRNA levels of* mdm2* remained unchanged during late torpor when p53 was activated. Interestingly, the protein levels of MDM2 were upregulated during early arousal by ~1.5-fold; this could potentially be attributed to the small (nonsignificant) elevation of* mdm2* mRNA levels during ET and LT compared to the control (Figure 4(a)). The selective increase of p53 target genes during transcriptional activation suggests that the affinity of p53 to different promoters of downstream targets is varied, and unique coactivators are likely required for transcription of different target genes during p53 activation. During the arousal stages, the elevated expressions of* 14-3-3, p21,* and* gadd-45α* observed during late torpor were normalized back to the control levels. Presumably, the reductions in mRNA levels were caused by the decrease in expression of p53 proteins from late torpor to arousal, resulting in the restabilization of p53 downstream gene expressions. The fold induction of p53 downstream target genes observed in this study was within the range of 1.6- to 1.75-fold, although this may be considered minor; previous studies have shown that stabilization of p53 by apigenin, a flavonoid that inhibits UV-induced skin tumorigenesis in mice, results in a small but important 1.5- to 2-fold increase in p21 expression [28]. Furthermore, earlier studies have shown that a slight increase in cell cycle inhibitor p16 by 1.5-fold can have significant impact on cancer resistance [29, 30].

Skeletal muscles can undergo atrophy during prolonged periods of disuse or immobilization; intriguingly, hibernating mammals are protected against normal muscle mass loss despite long periods of inactivity during torpor. Although hibernators are resistant towards muscle atrophy, a study by Andres-Mateos et al. shows that muscle regeneration in hibernating mammals after tissue damage is actually delayed compared to activate squirrels and suggests that muscle differentiation is inhibited during torpor until 6–8 weeks after injury [31]. The activation of p53 protein has recently been shown to repress the transcription of myogenin (myod), a myogenic regulatory factor that plays a critical role in muscle differentiation [32]. It was proposed that repression of myogenin by p53 likely serves to prevent aberrant differentiation or proliferation of myogenic cells during unfavourable/stressful cellular environment for which p53 is activated. The activation of p53 during torpor observed in our study could also function in a similar manner to negatively regulate muscle differentiation during torpor and is consistent with our previous observations of reduced* myod* mRNA levels during torpor [33]. The activation of p53 could serve as a potential signaling mechanism to delay nonessential skeletal muscle differentiation during torpor that would allow redirection and prioritization of energy expenditure to support functions of vital tissues such as the heart and the brain during hibernation.

**Figure 5:**
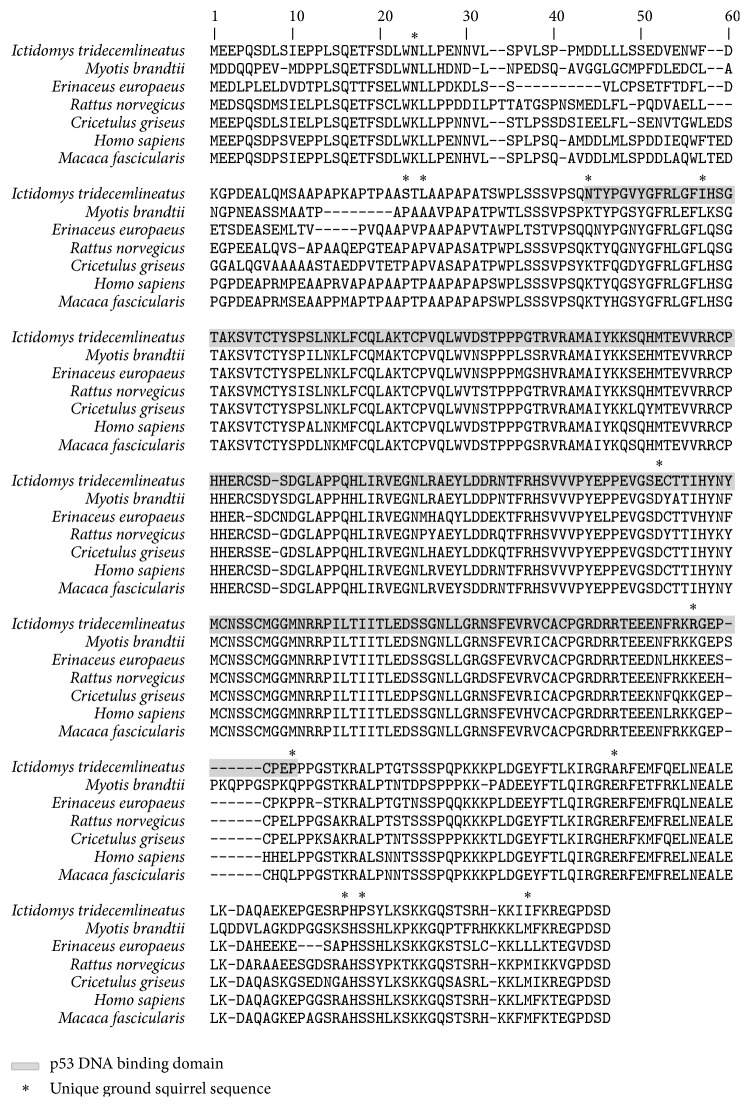
Sequence comparison of the ground squirrel p53 protein. Amino acid alignments of the ground squirrel p53 protein with other hibernating and nonhibernating mammals. *∗* denotes unique substitutions in the ground squirrels at amino acid residues that are conserved among other nonhibernating mammals; residues shaded in grey indicate p53 DNA binding domain.

Intriguingly, we observed two squirrel specific sequence substitutions at amino acid residues that are normally modified in human that signal p53 for degradation by MKRN1 and IKK2 (Figure 5) [19, 20]. MKRN1 is a transcriptional coregulator and an E3 ligase that targets p53 for degradation via recognition of the K292 amino acid residue. The K292 residue is highly conserved from* Caenorhabditis elegans* to human and is mutated in several different human cancers [19]. Mutations of p53 at K292 and a neighboring K291 residue highly increase p53 stability, with only a 14–18% degradation in presence of MKRN1 compared to a 70% reduction in the wild-type [19]. Meanwhile, phosphorylation of p53 by IKK2 at S366 leads to its ubiquitination by the *β*-TrCP1 F-box protein and is subsequently targeted for degradation independent of MDM2 [20]. The K292 and S366 are conserved amino acid residues that are required to promote p53 degradation in human and are both altered in the ground squirrel to residues that can no longer be acetylated (K292R substitution) or phosphorylated (S364P substitution). Other amino acid change of potential interest is the Lys→Asn substitution at position 24 in the ground squirrels. The K24N mutation of p53 has previously been reported in rare form of human gestational cancer; although this mutation increases the flexibility of p53 transactivation domain, it does not seem to significantly alter its interaction with MDM2 [34].

The cellular levels of p53 are typically determined by the rate at which it is degraded through the ubiquitin-mediated pathway, rather by the rate at which it is synthesized [35]. While MDM2 functions as the primary antagonist of p53 expression, our study shows that an increase in p53 expression alone in absence of a change in MDM2 protein levels can also result in the activation of p53 transcriptional activity. Although the functional significance of unique amino acid residues in ground squirrels will require further analysis to determine its functions, it can be suggested that these substitutions may contribute to an overall increase in p53 stability during torpor when levels of MDM2 are not affected. Unique amino acid changes in hibernators have previously been reported and suggested as potential evolutionary adaptation methods to contribute to an altered phenotype. The insulin growth factor 1 receptor in the long-lived and hibernating* Myotis brandtii* bats contains unique amino acid substitutions at the highly conserved single-transmembrane domain, and this is thought to be associated with an altered expression of insulin-associated genes in the bats that is similar to those observed in the long-lived GHR^−/−^ mice [36]. Sequence variation in the glycerol-3-phosphate dehydrogenase (G3PDH) of ground squirrels has also been reported, with unique amino acid substitutions predicted to contribute to a less rigid structure compared to other mammals, which could account for greater thermostability of G3PDH enzymatic function across different temperatures [37].

## 5. Conclusions

In this study, we reported the activation of the p53 transcriptional response during the torpor stages in the 13-lined ground squirrels. Increase in p53 activity appeared to be independent of the select posttranslational modifications examined in this study but rather a result of an increased p53 protein expression. We also identified two amino acid substitutions in the ground squirrel p53 protein sequence that were unique compared to other nonhibernating mammals; the K290R and S364P substitutions are of particular interest as these two residues contribute to the ubiquitin mediated degradation of p53 in human. In conclusion, our results show that the activation of p53 transcriptional response is mediated by its upregulation at the transcriptional and translational level, and further experiments will be needed to elucidate the significances of the ground squirrel specific amino acid substitutions, and whether these alterations contribute to changes in p53 stability compared to nonhibernating mammals.

## Figures and Tables

**Table 1:** Unique substitutions found in the ground squirrel p53 protein sequence at amino acid residues that are conserved in nonhibernating mammals. Bracket indicates amino acid position in human.

Conserved amino acid	Squirrel specific amino acid	Amino acid position	Property substitution
Lys	Asn	24	Charged → neutral
Pro	Ser	78 (80)	Nonpolar → polar
Pro	Leu	80 (82)	Conserved
Lys	Asn	99 (101)	Charged → neutral
Leu	Ile	112 (114)	Conserved
Asp	Glu	226 (228)	Conserved
Lys	Arg	290 (292)	Conserved
Leu	Pro	299 (302)	Conserved
Glu	Ala	334 (336)	Charged, polar → neutral, nonpolar (hydrophobic)
Ala	Pro	362 (364)	Conserved
Ser	Pro	364 (366)	Polar → nonpolar
Met	Ile	382 (384)	Conserved
